# Extreme Exposure to Filtered Far‐UVC: A Case Study^†^

**DOI:** 10.1111/php.13385

**Published:** 2021-02-03

**Authors:** Ewan Eadie, Isla M. R. Barnard, Sally H. Ibbotson, Kenneth Wood

**Affiliations:** ^1^ Scottish Photobiology Service Photobiology Unit NHS Tayside Ninewells Hospital and Medical School Dundee UK; ^2^ SUPA School of Physics & Astronomy University of St Andrews St Andrews UK; ^3^ Scottish Photobiology Service Photobiology Unit University of Dundee Ninewells Hospital and Medical School Dundee UK

## Abstract

Far‐UVC devices are being commercially sold as “safe for humans” for the inactivation of SARS‐CoV‐2, without supporting human safety data. We felt there was a need for rapid proof‐of‐concept human self‐exposure, to inform future controlled research and promote informed discussion. A Fitzpatrick Skin Type II individual exposed their inner forearms to large radiant exposures from a filtered Krypton‐Chloride (KrCl) far‐UVC system (SafeZoneUVC, Ushio Inc., Tokyo, Japan) with peak emission at 222 nm. No visible skin changes were observed at 1500 mJ cm^−2^; whereas, skin yellowing that appeared immediately and resolved within 24 h occurred with a 6000 mJ cm^−2^ exposure. No erythema was observed at any time point with exposures up to 18 000 mJ cm^−2^. These results combined with Monte Carlo Radiative Transfer computer modeling suggest that filtering longer ultraviolet wavelengths is critical for the human skin safety of far‐UVC devices. This work also contributes to growing arguments for the exploration of exposure limit expansion, which would subsequently enable faster inactivation of viruses.

## Graphical abstract

Far‐UVC is being heavily promoted as an important tool for the inactivation of SARS‐CoV‐2 in occupied spaces. While there is extensive laboratory evidence of efficacy and safety, there is little real‐world information on whether this technology is “safe for humans.” In this study, a single individual is exposed to extreme doses of filtered far‐UVC radiation, several hundred times the current exposure limit values. At the highest dose, (approx. 780 times the limit value) transient coloring was observed but no visible reaction was seen at 65× the exposure limit. Filtering longer wavelengths appears important for safety of this technology.

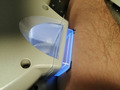

## INTRODUCTION

The severe acute respiratory syndrome coronavirus 2 (SARS‐CoV‐2) is the virus responsible for the current global COVID‐19 pandemic. Estimates as of the 4^th^ of December 2020 indicate 65.6 million confirmed coronavirus cases and approximately 1.5 million deaths globally (https://www.worldometers.info/coronavirus/). As of May 2020, the pandemic had also resulted in an estimated 3.8 trillion dollars of global consumption losses and 147 million job losses (1). As a consequence, it is imperative to employ measures that inactivate or destroy the virus and limit its transmission.

Ultraviolet‐C (UVC) radiation covers the wavelength range of 100–280 nm and has a known germicidal effect (2). UVC irradiation is a well‐established technology used for the destruction of bacteria and viruses and employed in a range of industries (3, 4, 5, 6). The established UVC wavelength routinely used for germicidal tasks is the mercury emission wavelength of 253.7 nm, which has been shown to inactivate SARS‐CoV‐2 but also results in acute adverse reactions in the skin and eyes (7, 8).

Far‐UVC is a term, which loosely incorporates wavelengths between 200 and 230 nm. Current far‐UVC published research is dominated by Krypton‐Chloride (KrCl) excimer lamps, which emit predominantly at 222 nm but can include low‐power long‐wavelength emissions. It has been demonstrated that far‐UVC, emitted by KrCl excimer lamps, inactivates SARS‐CoV‐2 on surfaces as well as human coronaviruses alpha HCoV‐229E and beta HCoV‐OC43 in air (9, 10). However, it does not induce premutagenic DNA lesions in mouse skin, even when chronically irradiating mice particularly susceptible to ultraviolet radiation (11, 12, 13). These laboratory data are being used commercially to intensively promote and sell far‐UVC systems to the global public. At the beginning of the COVID‐19 pandemic, the only published study investigating a far‐UVC system in humans had contradicted the laboratory results, showing skin damage in the form of erythema and cyclobutane pyrimidine dimer (CPD) formation (14). The authors of this study hypothesized that it may be longer wavelengths present in the lamp spectrum that caused the adverse effects, a hypothesis supported by subsequent computer modeling (15).

Due to the unsupported but widely disseminated commercial claims of far‐UVC systems being “safe for humans,” it was felt that there was a need for rapid proof‐of‐concept testing on human skin with an appropriately filtered far‐UVC device. This proof‐of‐concept testing could then inform future detailed and controlled assessment.

## MATERIALS AND METHODS

### In‐vivo exposure

A 37‐year‐old male, Fitzpatrick Skin Type II, performed multiple self‐exposures with a filtered KrCl excimer far‐UVC system with a peak wavelength emission at 222 nm (SafeZoneUVC, Ushio Inc., Tokyo, Japan). 5 × 5 cm areas on the left and right inner forearms were exposed on several occasions for exposure times of 250, 1000, 2000 and 3000 s. Exposure sites were assessed visually and with a reflectance spectrophotometer (CM‐700d with 8 mm aperture, Konica Minolta Inc., Tokyo, Japan) at hourly intervals from zero up to twelve hours and at 24 h. With one exposure (2000 s), the skin was tape stripped 1 h after exposure. On yet another exposure (2000 s), a second set of filtering was introduced to the far‐UVC source to further reduce the low‐power long‐wavelength emissions. All exposure areas were covered between time point assessments. The reflectance spectrophotometer output provides three values which represent L* (lightness from black to white), a* (from green to red) and b* (from blue to yellow) from the 1976 CIELAB color space. The effect of irradiation on skin redness was determined by calculating Δa the difference in a* at a given time point, a*(t), compared with preirradiation, a*(0). An increase in Δa represents an increase in redness. Similarly, Δ*b* is the effect of irradiation on skin “yellowness,” where an increase in Δ*b* represents an increase in yellow coloring (16).

The irradiance of the filtered far‐UVC source was determined with a broadband radiometer (International Light IL1400A meter with SEL220 sensor, QNDS2 filter and quartz diffuser. International Light Technologies, MA) and the spectral distribution with a double‐grating spectroradiometer (IDR300, Bentham Instruments Ltd, Reading, UK). The broadband radiometer was calibrated against the double‐grating spectroradiometer, which is itself calibrated against both a deuterium and quartz halogen tungsten lamp with traceability to national standards.

### Monte Carlo Radiative Transfer (MCRT) computer modeling

MCRT codes, previously used to study an unfiltered far‐UVC device, were used to investigate depth penetration of light from a filtered far‐UVC source as utilized in the self‐exposure (15). Optical properties of the skin layers and structure of the 5‐layer skin model were as previously described (15, 17). Results between filtered and unfiltered far‐UVC sources were compared.

## RESULTS

### In‐vivo exposure

**Figure 1:**
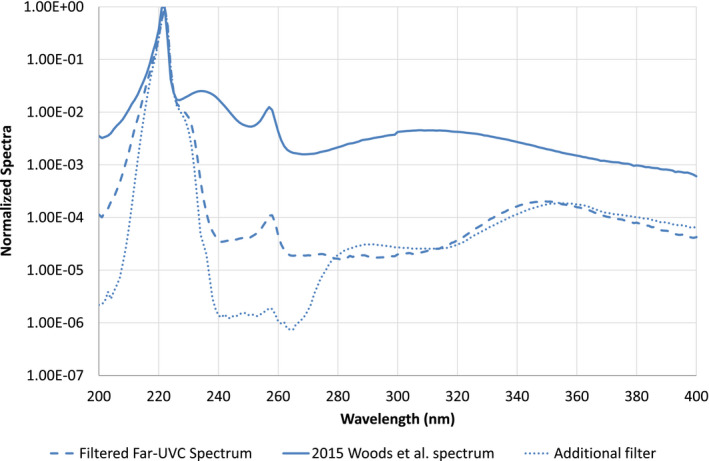
Normalized spectral distribution of the filtered far‐UVC source used in the majority of self‐exposures (dashed) and with additional filtering (dot). For comparison, the unfiltered far‐UVC source used in the study by Woods *et al*. is also plotted (solid). There were no obvious visual changes in the skin observed between the filtered and additionally filtered exposures.

Average irradiance on the skin surface from the filtered far‐UVC source was 6.1 mW cm^−2^ in the wavelength range 200–400 nm. For the exposure with additional filtering, the average irradiance on the skin surface was 5.8 mW cm^−2^. The inner forearm exposures were 1500, 6000, 12 000 and 18 000 mJ cm^−2^. The normalized spectral distribution of the filtered far‐UVC source is presented in Fig. 1, along with the spectral distribution with additional filtering.

**Figure 2:**
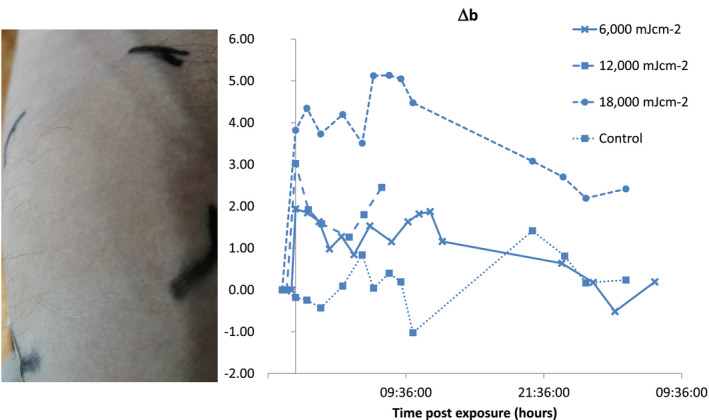
(Left) Right inner forearm following exposure to 12,000 mJ cm^−2^ filtered far‐UVC, 5 h post exposure. (Right) Change in irradiation site b* from the CIELAB color space at various radiant exposures. A positive value represents a yellowing of the skin.

There were no visible changes to the skin at a radiant exposure of 1500 mJ cm^−2^. However, exposures at or above 6000 mJ cm^−2^ resulted in a yellow coloring of the skin, which appeared immediately postirradiation and persisted for several hours (Fig. 2). Higher irradiations resulted in larger changes in color which persisted for longer. No erythema (redness) was evident at any time point, either visually or by reflectance measurement (i.e. no change in Δ*a*).

**Figure 3:**
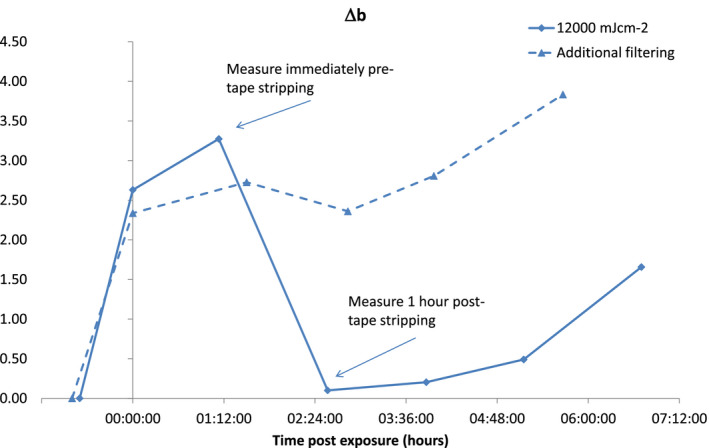
Change in irradiation site b* from the CIELAB color space. A positive value represents a yellowing of the skin. Persistent yellowing of the skin is observed even with the additional filtering displayed in Fig. 1. Tape stripping removes the yellow color from the skin.

Tape stripping of the skin, which removes the stratum corneum, initially reduced the yellow coloring suggesting that the changes in the skin were limited to the upper‐most superficial layers (Fig. 3). Additional filtering, to reduce the low‐power long‐wavelength emissions between 230 and 280 nm even further, appeared to have no effect on the yellow coloring.

### MCRT computer modeling

**Figure 4:**
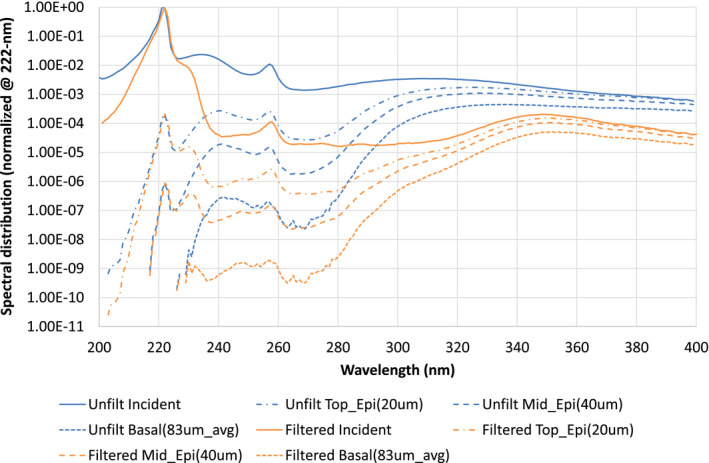
Results from Monte Carlo radiative transfer (MCRT) modeling of the filtered far‐UVC source (orange). The relative spectral fluence rate at selected depths within the skin is presented. These are compared with the MCRT modeling of Barnard *et al*. (blue) (15). Unfilt, Unfiltered, Epi, Epidermis.

Figure 4 details the fluence rate incident on different layers within the epidermis as defined by the MCRT computer modeling. There is roughly 100 times less incident on the basal layer between 240 and 320 nm, when comparing the filtered far‐UVC to the unfiltered source.

## DISCUSSION

These self‐exposure results have indicated that large radiant exposures (“doses”) of 1500 mJ cm^−2^ of filtered far‐UVC can be delivered to pale skin without induction of visible changes. Based on the results of Buonanno *et al*., such a dose within an 8 h limit would allow for an approximately 99.9% inactivation of airborne human coronavirus alpha HCoV‐229E in less than 1 min (9). Similarly, SARS‐CoV‐2 on a surface could undergo a 99.7% inactivation in less than 1.5 min (10). A dose of 1500 mJ cm^−2^ is much larger than the 23 mJ cm^−2^ limit of exposure in the International Commission on Non‐ionizing Radiation Protection (ICNIRP) guidelines (18). The ICNIRP limit of exposure represents “conditions under which it is expected that nearly all individuals may be repeatedly exposed without acute adverse effects and, based upon best available evidence, without noticeable risk of delayed effects” (18). This proof‐of‐concept study in no way replaces these guidelines and associated national legislations but is a baseline for further explorative, controlled research studies. The ICNIRP limits of exposure also apply to the eye, which this study has not investigated.

At much larger doses, 6000 mJ cm^−2^, of filtered far‐UVC, a reaction in the skin was observed, with coloring appearing immediately post exposure. This pattern is similar but not identical to immediate pigment darkening (IPD), which is the photo‐oxidation of existing melanin, routinely seen with exposure to ultraviolet‐A (UVA) and UVA1 radiation. However, several factors indicate that the coloring observed is not photo‐oxidation of existing melanin. Firstly, the small UVA radiant exposure from the filtered far‐UVC lamp is much lower than would normally be required to induce IPD. Secondly, the color observed is different in appearance to that normally observed for IPD, with the tape stripping indicated that most of the color change is confined to the stratum corneum. Finally, introducing additional filtering to reduce longer wavelengths made no difference to the color change which indicates that it is the primary 222 nm wavelength causing the color change and not longer wavelength emissions.

In the 2015 study by Woods et al., the Minimal Erythema Dose (MED) from exposure to the unfiltered far‐UVC device was 40–50 mJ cm^−2^; whereas, in the current report, no erythema was observed with the filtered far‐UVC device self‐exposure of 1500 mJ cm^−2^ (or up to 18 000 mJ cm^−2^). This difference would support the hypothesis from Woods et al., and subsequently reinforced by Barnard *et al*., that longer ultraviolet wavelengths were responsible for the skin damage seen in the 2015 Woods et al. study (14, 15). This is proven further in a recent publication by Buonanno *et al*. (19). It is well recognized that it is important, when assessing the hazard from an ultraviolet source, to consider all wavelengths and plot the source spectrum on a logarithmic scale (20).

A study by Fukui *et al*. found similar results to our self‐exposure, with no visible erythema at 24 h following 500 mJ cm^−2^ irradiation with a filtered far‐UVC device (21). That study also reported on higher cyclobutane pyrimidine dimers (CPD) in the irradiated region compared with a control site, although the analysis used was not able to determine in which section of the skin the CPDs occurred. We have addressed this gap in knowledge in a recent rapid communication, which indicates that CPD induced by filtered far‐UVC (radiant exposure 6000 mJ cm^−2^) is restricted to the supra‐basal layers of the skin (22). Our MCRT computer modeling supports both these studies as we demonstrate all wavelengths, including 222 nm, can penetrate to the top and middle of the epidermis (Fig. 4). In addition, our previous study also demonstrated that CPD can be induced by all wavelengths, including 222 nm, in the upper and midepidermis (15). Therefore, we propose that the CPD observed by Fukui et al. and Hickerson et al. are likely to have occurred in the upper epidermis where it is thought that DNA damage will not lead to induction of skin cancer (21, 22).

This single individual study does not provide a definitive answer to the question of skin safety. Our study is the basis for future exploration above the current ICNIRP limit values, which would allow quicker inactivation of the virus than is currently permitted in occupied spaces. Furthermore, what this research and other published literature clearly highlight is that the hazard of all wavelengths emitted must be appropriately assessed—it is too simplistic to state that far‐UVC devices are “safe for humans.”
